# Prevalence of dementia and major dementia subtypes in Spanish populations: A reanalysis of dementia prevalence surveys, 1990-2008

**DOI:** 10.1186/1471-2377-9-55

**Published:** 2009-10-19

**Authors:** Jesús de Pedro-Cuesta, Javier Virués-Ortega, Saturio Vega, Manuel Seijo-Martínez, Pedro Saz, Fernanda Rodríguez, Angel Rodríguez-Laso, Ramón Reñé, Susana Pérez de las Heras, Raimundo Mateos, Pablo Martínez-Martín, José María Manubens, Ignacio Mahillo-Fernandez, Secundino López-Pousa, Antonio Lobo, Jordi Llinàs Reglà, Jordi Gascón, Francisco José García, Manuel Fernández-Martínez, Raquel Boix, Félix Bermejo-Pareja, Alberto Bergareche, Julián Benito-León, Ana de Arce, José Luis del Barrio

**Affiliations:** 1 National Centre for Epidemiology, CIBERNED and Alzheimer' Disease Research Unit, Carlos III Institute of Public Health, Madrid, Spain; 2 Primary Care Administration, Segovia, Spain; 3 Neurology Unit, Salnés Hospital, Pontevedra, Spain; 4 Department of Medicine and Psychiatry, Zaragoza University, Zaragoza, Spain; 5 Neurology Unit, Segovia Hospital, Segovia, Spain; 6 Madrid Regional Health Authority, Madrid, Spain; 7 Dementia Diagnosis and Treatment Unit, Neurology Department, Bellvitge University Teaching, Spain; 8 Llodio Health Center, Day Medical Center, Oroityu-Getxo-Vizcaya, Spain; 9 Psychiatry Department, University of Santiago de Compostela, Spain; 10 Neurology Unit, Virgen del Camino Hospital, Pamplona, Spain; 11 Dementia Unit. Hospital Santa Caterina, Institut d'Assistència Sanitària, Salt, Spain; 12 Barcelona Hospital, Barcelona, Spain; 13 Geriatrics Unit. Virgen del Valle Geriatric Hospital., Toledo, Spain; 14 Neurology Service, Cruces Hospital, Day Medical Center, Oroityu-Getxo-Vizcaya, Spain; 15 Neurology Department, 12 de Octubre University Teaching Hospital, Madrid, Spain; 16 Centro de Investigación Biomédica en Red sobre Enfermedades Neurodegenerativas (CIBERNED), Madrid, Spain; 17 Neurology Department, Bidasoa-Hondarribia Hospital, Guipúzcoa, Spain

## Abstract

**Background:**

This study describes the prevalence of dementia and major dementia subtypes in Spanish elderly.

**Methods:**

We identified screening surveys, both published and unpublished, in Spanish populations, which fulfilled specific quality criteria and targeted prevalence of dementia in populations aged 70 years and above. Surveys covering 13 geographically different populations were selected (prevalence period: 1990-2008). Authors of original surveys provided methodological details of their studies through a systematic questionnaire and also raw age-specific data. Prevalence data were compared using direct adjustment and logistic regression.

**Results:**

The reanalyzed study population (aged 70 year and above) was composed of Central and North-Eastern Spanish sub-populations obtained from 9 surveys and totaled 12,232 persons and 1,194 cases of dementia (707 of Alzheimer's disease, 238 of vascular dementia). Results showed high variation in age- and sex-specific prevalence across studies. The reanalyzed prevalence of dementia was significantly higher in women; increased with age, particularly for Alzheimer's disease; and displayed a significant geographical variation among men. Prevalence was lowest in surveys reporting participation below 85%, studies referred to urban-mixed populations and populations diagnosed by psychiatrists.

**Conclusion:**

Prevalence of dementia and Alzheimer's disease in Central and North-Eastern Spain is higher in females, increases with age, and displays considerable geographic variation that may be method-related. People suffering from dementia and Alzheimer's disease in Spain may approach 600,000 and 400,000 respectively. However, existing studies may not be completely appropriate to infer prevalence of dementia and its subtypes in Spain until surveys in Southern Spain are conducted.

## Background

Spain is one of the fastest aging societies in the world. According to the aging index, the proportion of the Spanish population aged 65 years and over (16.8% in 2004) have doubled in the last 30 years ranking seventh among European countries . Therefore, the burden of chronic neurodegenerative disorders, particularly dementia, is expected to grow exponentially.

Dementia screening surveys are population-based studies in which individuals that are screened positively through a cognitive or disability test, undergo a second phase of clinical assessment and diagnosis. The screening survey is the only methodologically sound research design capable of estimating dementia prevalence. It copes effectively with bias due to underdiagnosis of dementia in the community.

A review by the *Spanish Epidemiological Study Group on Aging *identified reports on age- and sex-specific prevalence of dementia from screening surveys referred to 6 populations conducted from 1990 to 2000 [1]. Crude dementia prevalence for elders aged 70 years or more ranged from 6.6% in Zaragoza to 17.2% in Pamplona. Prevalences of Alzheimer's disease (AD) in these surveys were 5.1% and 10.6%, respectively. Vascular dementia (VD) was the second most frequent dementia subtype [1]. However, prevalence reported for Spanish populations by 2005 might be limited given the methodological differences across studies and the recent improvements in diagnostic criteria for infrequent dementia subtypes. In addition, the above-mentioned surveys focused on populations resident in the Northern half of the country, while Southern populations remain under-reported. The latter might be particularly important given the higher vascular mortality found in Southern Spain and the impact of vascular factors in the etiology of dementia [2].

This study sought to 1) estimate the prevalence of dementia in Spain reanalyzing the data from published and unpublished dementia prevalence screening surveys conducted to date, 2) identify study features related to the magnitude of dementia prevalence, and 3) compare the prevalence of dementia found in Spanish studies with that of selected European surveys.

## Methods

In this report, we followed the recommendations of the Meta-analysis of Observational Studies in Epidemiology Group [3] for the purposes of identifying, appraising, synthesizing, and combining results from different surveys.

### Search strategy

Studies on dementia prevalence in Spain were searched up to December 31, 2008 in PubMed, Indice Médico Español and Biblioteca Virtual en Salud. The following search words were used as MeSH or TIAB terms: "dementia," "prevalence," "door-to-door," "screening" and "Europe." Search was restricted to reports published in English and Spanish within the period 1985 through 2008. Search yielded 23 reports in English and 3 in Spanish. Upon authors' request, the Spanish Society of Neurology and the Spanish Society of Epidemiology provided information about one ongoing survey in Murcia (Eastern Spain).

### Studies selection

Inclusion and exclusion criteria for survey selection approved by consensus by the *Spanish Epidemiological Study Group on Aging *were as follows: 1) survey incorporates an updated population census for a study population geographically defined by residence, 2) survey uses a screening instrument in the first phase of the study, 3) survey describes the diagnostic methods and the medical specialist in charge of diagnosis (e.g., geriatrician, neurologist, psychologist); 4) survey uses specific diagnostic criteria for dementia subtypes; and 5) survey includes no less than 20 dementia cases. Inclusion and exclusion criteria assured a minimum quality threshold.

Surveys potentially valid for analysis finished data collection by October 2008. The pool of potentially valid surveys included 1 pilot study [4], 14 published surveys [2-19], and 1 unpublished survey [20]. The target populations of these studies were: Pamplona, Zaragoza-ZARADEMP (two-time point measurements), NEDICES (three geographically distinct sub-populations in Central Spain), Toledo, Leganés, Gerona, La Selva del Camp, Turégano, Alcoi/Bañeres, Bidasoa and Munguialde, El Prat de Llobregat and Murcia.

Inclusion criteria were independently rated in each pre-selected survey by three co-authors (RB, JLB and JPC) through a questionnaire for data-collection, focusing on demographic, methodological, diagnostic, disability-related and epidemiologic data to be requested from the authors of the selected surveys [5-11,13-21]. The NEDICES survey and studies in La Selva del Camp and Alcoi/Bañeres [8,13,14] were excluded due to incomplete dataset. Authors of the ongoing study in Murcia [20] refrained from participating before the publication of their original data. Data from ZARADEMP first measurement was not provided [21]. The pilot study by Bermejo-Pareja [4] and a study reporting 11 dementia cases aged ≥ 65 years [12] were excluded due to small sample size.

Nine studies covering 9 geographically different populations met inclusion criteria: Bidasoa, Munguialde, Gerona, Leganés, Pamplona, El Prat de Llobregat, Toledo, and Zaragoza, ZARADEMP one population each [5-7,9-11,15-17,19]. Since some studies solely covered populations aged 70 years and over, the sub-populations used for reanalysis of selected studies comprised subjects over the age of 69 years, divided into 4, 5-year sex-specific strata, and two open-ended groups aged ≥ 70 and ≥ 90 years respectively.

**Figure 1:**
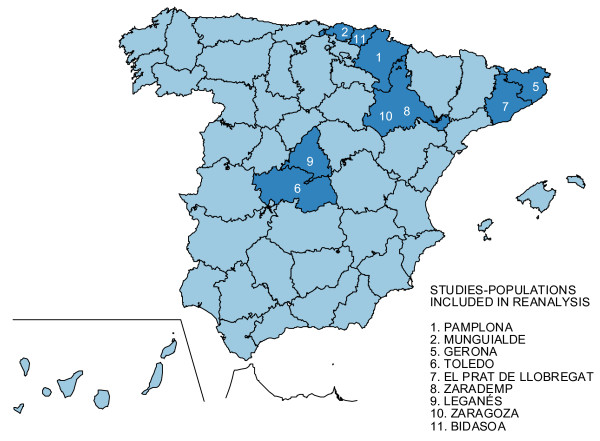
**Geographical location of reported and not reported, reanalyzed dementia prevalence surveys in Spain**.

The methodological characteristics of the selected surveys are presented in Additional File 1. The geographical locations of the surveys covered by reanalysis are shown in Figure 1. Prevalence years ranged from 1990 to 2005. The population size ranged from 524 in Leganés to 2,850 in ZARADEMP, and the number of cases went from 48 in Bidasoa to 214 in ZARADEMP. Data on the size of the municipalities where the participants lived was obtained from the National Institute of Statistics . Municipalities were classified as rural (<2,000 inhabitants) and urban (≥ 10,000 inhabitants). Urban metropolitan populations with a high proportion of immigrants, i.e., born outside the municipal boundaries, were denoted as suburban. An urban mixed category was used to identify former rural populations which had become urban in recent decades.

### Assessment of prevalence variation within Spanish reanalyzed surveys

Age- and age- and sex-specific prevalence of dementia and major dementia subtypes were obtained for four 5-year age groups and an open ≥ 90 years group. Crude prevalence proportions, as well as age- and sex-adjusted prevalence were calculated for the population aged ≥ 70 years. We used the European standard population as the basis for adjustment (weights 0.43, 0.29, 0.14 and 0.14 for 75-79, 80-84 ad 85 and over years, respectively; .

For the purposes of assessing prevalence of dementia and its major subtypes, data from reanalyzed surveys were weighted against those of El Prat, which was taken as reference. El Prat was selected as reference due to its recency and large study size [17]. Unconditional logistic regression on grouped data controlling for age and sex was conducted. The prevalence variation in reanalyzed surveys by environment (rural, urban), diagnosing specialists (psychiatrist, neurologist, geriatricians) and percentage of participation of targeted population, was assessed using the same multivariate methods. Diagnosing specialist was considered a predictive factor due to the different diagnostic patterns among specialists with different backgrounds [19]. All analyses were carried out with Stata v. 8.0 (Stata Corporation, College Station, Texas).

## Results

**Figure 2:**
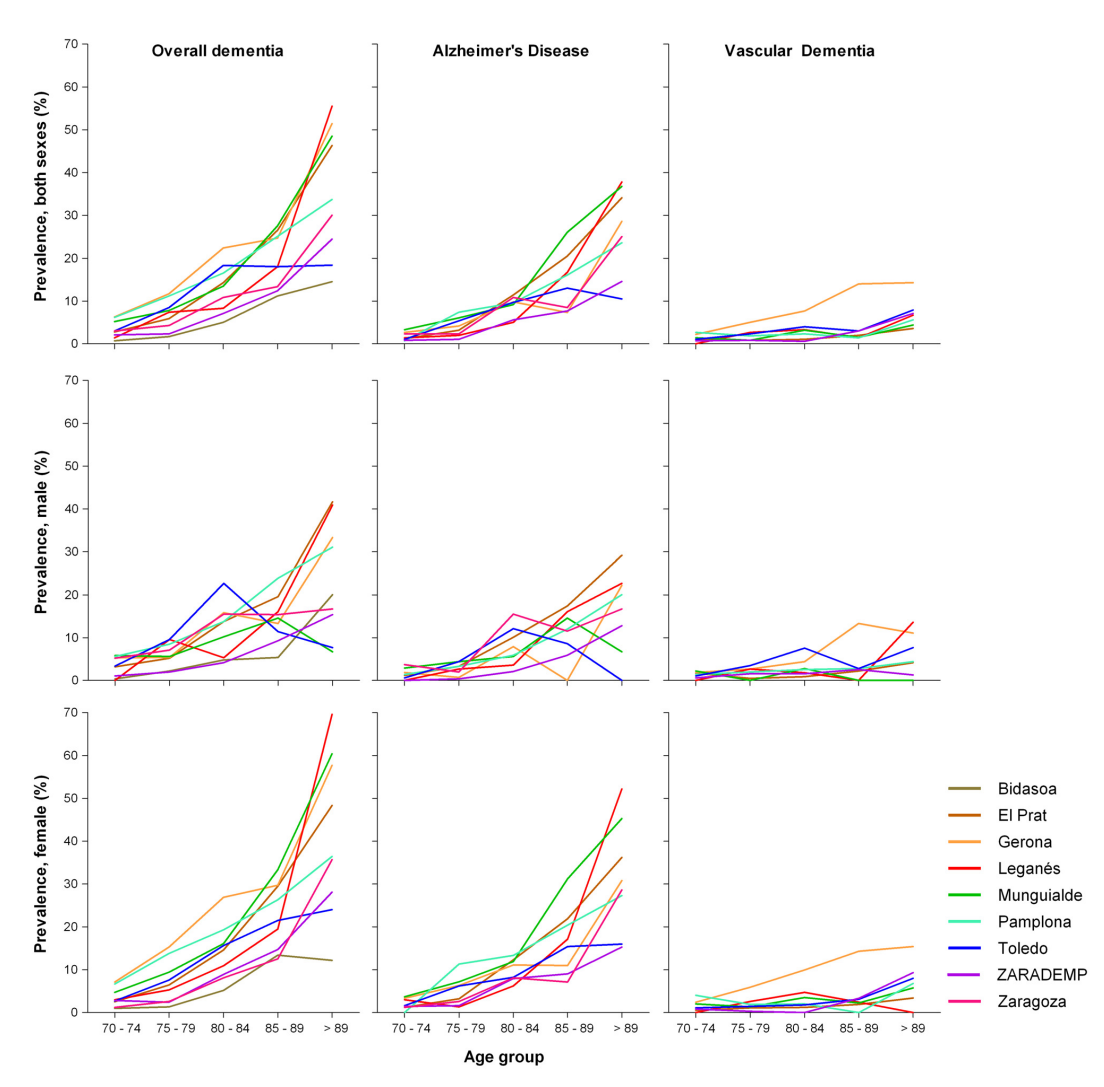
**Age-specific prevalence of dementia, Alzheimer's disease and vascular dementia in reanalyzed Spanish populations by gender**.

The combined population for reanalysis, hereafter Spanish-reanalyzed population, totaled 12,232 persons (7,150, 58% women) and yielded 1,194 cases of dementia; 707 AD cases, and 238 cases of VD. The Spanish-reanalyzed population came from North and Central Spain and varied in terms of living environment. For instance, while the Zaragoza, Gerona, and Pamplona surveys focused on urban populations, those in Leganés and El Prat were suburban with a high proportion of immigrant population (~86%). Other sub-populations were less homogeneous, e.g., the Bidasoa and Toledo areas had a high proportion of immigrants and were both classified as urban-mixed. Detailed age- and sex-specific prevalence of dementia, as well as crude, and age- and sex-adjusted counts for participants aged ≥ 70 years are shown in Additional File 2. Prevalence estimates of 9 and 8 sub-populations were available for AD and VD respectively. Age- and sex-specific prevalences are presented in Figure 2.

Dementia prevalence varied substantially across surveys in age-specific groups: up to four-fold increase among the oldest group (Figure 2). Differences in crude prevalence for participants aged ≥ 70 years were consistent across gender and age groups (Figures 2), ranging from 3.5% in Bidasoa to 17.2% in Pamplona. Age- and sex-adjusted prevalence in the same age-group ranged from 3.2% in Bidasoa to 12.3% in Gerona, while the corresponding figures in the El Prat survey were intermediate (9.6 and 9.3%).

In the case of AD both sexes, age- and sex-adjusted prevalence at age ≥ 70 years, ranged from 2.6% in ZARADEMP to 7.7% in El Prat, and in the case of VD, from 1.2% in El Prat and ZARADEMP to 5.1% in Gerona.

Insofar as Lewy body dementia (LBD) and frontotemporal dementia (FTD) were concerned, little information was available in the Spanish-reanalyzed population. Crude LBD prevalence at ≥ 70 years was 2.0% in El Prat and Bidasoa and 0.8%, including parkinsonism with dementia in Muguialde. The corresponding values for FTD were 2.0% (2 cases) in El Prat, 0.1% in Munguialde (1 case).

The common age- and sex-specific pattern for all surveys can be summarized as follows: 1) an increase in dementia prevalence with age across sex groups, higher for AD than for VD; and, 2) a higher prevalence of dementia among women (Figure 2).

The logistic regression results (Additional File 3) pointed to a significantly higher prevalence of dementia in women OR 1.45 (95%CI 1.27 - 1.66), increasing with age and differing by place of residence. The prevalence of dementia in both sexes in El Prat proved significantly higher than that in some of the reanalyzed surveys (ZARADEMP, Zaragoza and Bidasoa). The overall OR variation between Gerona and Bidasoa was five-fold. Alzheimer's disease prevalences of Spanish-reanalyzed sub-populations referred to El Prat showed significant departures in two surveys: Bidasoa, OR = 0.42 (95% CI 0.32 - 0.55), and Pamplona, OR = 1.35 (95% CI 1.03 - 1.77). For VD, with few populations available for comparison, differences were significant when Gerona, OR = 5.00 (95% CI 3.06 - 8.16), and Toledo, OR = 2.05 (95% CI 1.12 - 3.73), were considered. ORs for the oldest age group showed a seven-fold and eighteen-fold increase for VD and AD respectively.

Results of reanalysis by type of diagnosing specialist, living environment and level of participation are reported in Additional File 4. Lower prevalence of AD, VD and overall dementia were reported by surveys carried out by psychiatrists compared to neurologists and geriatricians. Similar prevalence of AD and higher prevalence of VD among men were found when diagnosis was conducted by geriatricians as opposed to neurologists. Reanalyzed suburban populations of both sexes had a lower VD prevalence, and higher prevalence of AD than urban sub-populations did. Significantly lower prevalences of dementia, AD and VD were systematically found when participation was below <85%. The only unreported survey analyzed (Bidasoa) showed lower dementia prevalence when weighted against published surveys, OR = 0.32 (95% CI 0.24 - 0.44).

## Discussion

The present study is a comprehensive, comparative approach to the prevalence of dementia and its subtypes in Spanish populations. The most relevant findings can be summarized as follows: 1) dementia prevalence was highly variable across reanalyzed sub-populations, 2) dementia prevalence attributable to AD is higher among women; 3) dementia prevalence varied as a result of the composition of the diagnostic team (neurologist, psychiatrist, geriatrician, psychologist), the municipality category (urban, suburban, urban-mixed) and the percentage of participation of originally screened participants. Inconsistent results across studies may be whether due to the diverse designs used across studies or due to actual prevalence variations caused by changes in incidence or survival. Since there are limitations due to study power, these results should be taken cautiously.

Although the present study incorporated three surveys not previously reanalyzed [1], it lacked information on the prevalence of dementia in Southern Spain. This may be particularly misleading given that cardiovascular and cerebrovascular mortality is reportedly higher in Southern Spain [2,22]. Moreover, prevalence of vascular risk factors (low folic acid intake, hypercholesterolemia, arterial hypertension, diabetes mellitus and smoking habit) is also higher in this region [23,24]. Unfortunately, surveys conducted in areas where a high mortality due to stroke have been reported (South-West Spain (Alcoi/Bañeres and Murcia) [22-25] could not enter the analysis.

Since variation in screening instruments and participation level were modest (range 62%-92.4%), it could be argued that differences in prevalence of dementia were largely caused by inconsistent methods across surveys. Leganés study [10] implemented sequential screening methods that may have induced losses at follow up. On the other hand, Leganés and Toledo studies [9,10] may have shown an artifactually high sensitivity resulting from the clinical evaluation of a large group of negatively screened participants. However, these shortcomings may have not been highly detrimental to the internal validity of these studies since the prevalence reported for Leganés and Toledo fell within the range of variation of other surveys (see Figure 2).

Regarding specialist-specific diagnostic patterns, although there is some evidence suggesting that psychiatrist approach dementia differently as opposed to other medical professionals [26], our findings may be considered artifactual. First, there is only one study involving a single geriatrician, and he happened to be assisted by a psychiatrist [9]. Therefore specific diagnostic patterns for geriatricians and psychiatrists can not be clearly disentangled. Second, the seemingly high likelihood of dementia diagnosis associated to geriatricians was limited to vascular dementia (Additional File 4). Third, Toledo is located in the mid-South of the country were cardiovascular mortality has been found to be higher [2], which suggests further that differences found in Toledo may not be solely attributable to the background of the diagnosticians. In summary, it suffices to say that variation in the background of diagnostic teams across studies may have induced some degree of heterogeneity to dementia prevalence. However, the limited number of studies and the various compositions of diagnostic teams preclude any specific conclusion as regards to univocal relationships between a given specialist and over- or under-diagnostic trends.

The impact of new entities in recent surveys, such as FTD or LBD in El Prat, or FTD in Bidasoa, may have replaced cases labeled as mixed dementia in earlier surveys, thus leaving the proportion of AD relatively unchanged. The high prevalence of VD reported in Gerona may be attributed to less restrictive diagnostic criteria [1,27-30].

**Figure 3:**
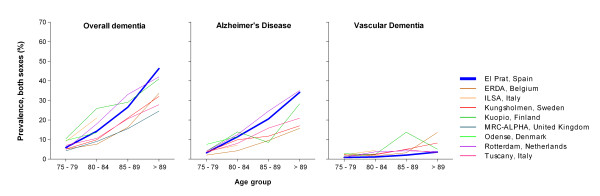
**Age-specific prevalence of dementia, Alzheimer's disease and vascular dementia in European and El Prat populations, data for both sexes**.

A visual comparison of selected age- and sex-specific prevalence reported in European populations [31-36] suggests that the variation of dementia prevalence in Spain may be higher (Figure 3). Again, differences may have been caused by the varying methodological features of the studies under analysis. Studies in El Prat [17] and Mungialde [18] showed lower prevalences of LBD in these Spanish populations when compared to the European populations of Kuopio (Finland) and Islington (UK) [37,38]. This could also be attributed to differing methods, particularly diagnostic standards used for LBD.

The number of dementia and AD patients in the Spanish population (age ≥ 70 years) based on El Prat prevalence was: 10.9% (95% CI 9.4% - 12.3%) and 608,000 cases (95%CI 526,000 - 689,000) for dementia; and 7.7% (95% CI 6.4% - 9.0%) and 431,000 cases (95%CI 359,000 - 502,000) for AD, respectively. However, these values, while similar to those found in other surveys, may not allow a valid extrapolation to the whole Spanish population.

Traditionally, differences in prevalence are attributed to methods, incidence, survival and migration [39]. The available evidence on incidence of dementia and its subtypes in Spanish populations is sparse, making it hard to attribute the differences observed to incidence variations. An exception that could well fit our findings is the study conducted in Girona, which offered 23.2 and 10.8 per 1000 person-years for dementia and AD, respectively, for those aged 75 years or more [27]. Regarding disease duration and survival of dementia subtypes, while differences exist in terms of higher mean duration of AD, lower mean duration of FTD (6.2 versus 4.2 mean years [40]), and shorter duration of dementia with clinical vascular components [41], it seems that the impact of these factors on prevalence is still difficult to estimate. Most studies show a slower increase of age-specific prevalence of VD with age as opposed to age-specific prevalence of AD, which may be attributable to shorter survival. Methodological aspects affecting under-diagnosis in Central Spain surveys have been pin-pointed by Bermejo et al. [42] and have been observed here, particularly those related to non-participation and screening validation. Such problems might account for the lower dementia prevalence of the unreported survey.

## Conclusion

Prevalence of dementia and AD in Central and North-Eastern Spain is higher in females, increases with age, and displays considerable geographic variation that may be method-related. Prevalence figures were similar or lower than those found in Western Europe. The present reanalysis indicated that some 600,000 and 400,000 people have dementia and AD in Spain, respectively. However, extrapolation should be taken cautiously until surveys conducted in Southern Spain are available.

## List of abbreviations

AD: Alzheimer's disease; VD: vascular dementia; LBD: Lewy body dementia; FTD: Fronto-temporal dementia. OR: Odds ratio; CI: Confidence Interval.

## Competing interests

The authors declare that they have no competing interests.

## Authors' contributions

JLB, JPC and RB participated in designing the reanalysis methods, and drafted the first manuscript. JLB and JVO participated in day-to-day coordination, managed the database, performed most of the statistical analysis, and contributed to later versions of the manuscript. JPC, FBP, ARL and most other authors participated in study design, data-collection, and in a critical review of the manuscript. JPC (study coordinator), PM, and FBP conceived the study. All authors read and approved the final manuscript.

## Pre-publication history

The pre-publication history for this paper can be accessed here:



## Supplementary Material

Additional file 1 **Supplemental Table S1**. Methodological aspects of selected, door-to-door prevalence surveys of dementia in Spanish populations. Click here for file

Additional file 2 **Supplemental Table S2**. Age and sex-specific counts for aged ≥ 70 years, crude, and age-adjusted or age- and sex-adjusted prevalence of dementia, Alzheimer's disease and vascular dementia in reanalyzed Spanish surveys. Click here for file

Additional file 3 **Supplemental Table S3**. Prevalence OR of dementia, Alzheimer's disease and vascular dementia with 95% CI, adjusted for age and age- and sex (reference: El Prat survey). Click here for file

Additional file 4 **Supplemental Table S4**. Age- and sex-adjusted prevalence odd ratios plus 95% confidence interval of dementia, Alzheimer's disease and vascular dementia for selected factors among ≥ 70 participants. Click here for file
