# Supplementary material for: Prevalence of dementia and major dementia subtypes in Spanish populations: A reanalysis of dementia prevalence surveys, 1990-2008
Source: BMC Neurol. 2009 Oct 19;9:55. doi: 10.1186/1471-2377-9-55 (PMC2770986; doi:10.1186/1471-2377-9-55)
Supplement: Additional file 1 — Supplemental Table S1. Methodological aspects of selected, door-to-door prevalence surveys of dementia in Spanish populations. [file 1471-2377-9-55-S1.DOC]

**Table 1:** Methodological aspects of selected, door-to-door prevalence surveys of dementia in Spanish populations.

.

| **Reference** | **Population**  **residence and habitat** | **No. individuals**  **(% response)^†^** | **Dementia,**  **no. cases** | **Age**  **(years)** | **Prevalence year** | **Screening phase**  **Instrument & Field workers** | **Diagnostic**  **ascertainment phase** |
| --- | --- | --- | --- | --- | --- | --- | --- |
| GERONA (11) | Gerona, urban | 1,460  (92.4%) | 200 | ≥ 70 | 1990 | MEC (*Mini Examen Cognitivo*), Spanish version of MMSE (Mini Mental State Examination). Cut-off: 23/24  Field workers: 18 previously trained general practitioners and nurses. | Clinical psychologist and neurologist.  CAMDEX for dementia and dementia subtypes. |
| PAMPLONA (7) | Pamplona (Navarre), urban | 1,127  (Not specified) | 194 | ≥ 70 | 1991 | CAMCOG (Cambridge Examination for Mental Disorders of the Elderly. Modified Spanish version: joint administration to patient and caregiver. Including the MMSE). Cut-off: < 74.  Field workers: three trained physicians. | Clinical psychologist and neurologist.  CAMDEX for dementia and dementia subtypes. |
| ZARAGOZA (5) | Zaragoza, urban | 815  (80%) | 54 | > 64 | 1992 | MEC (*Mini Examen Cognitivo*), Spanish version of MMSE. Cut-off: 23/24.  Field workers: 10 trained medical students. | 3 psychiatrists blind to result of screening phase I  DSM-III-R for dementia |
| TOLEDO (9) | Toledo, urban-mixed | 933  (81.7%) | 90 | > 64 | 1994 | Spanish version of MMSE from Folstein et al. Cut off: 17/18 for illiterates, 20/21 less than primary school and 23/24 primary school education.  Field workers: 17 trained psychologists | 1 geriatrician or 1 psychiatrist (for diagnosis of dementia) supported by 1 neurologist and 1 psychologist (CAMCOG).  CAMCOG Spanish version.  DSM-III-R for dementia. |
| ZARADEMP (6) | Zaragoza municipal area, urban | 2,850  (83%) | 214 | ≥ 55 | 1996 | MEC (*Mini Examen Cognitivo*), Spanish version of MMSE (Mini Mental State Examination). Cut-off: 23/24.  Field workers: trained interviewers | 3 psychiatrists blind to result of phase-I screening  DSM-IV for dementia |
| BIDASOA (15,16) | Irún, Hondarribia (Guipúzcoa), urban-mixed | 1,349  (84%) | 48 | ≥ 65 | 1996 | WHO, SNES modified  (Sicilian Neuro-Epidemiological Study) tasks and questionnaire  Clinical examination  Field workers: trained sociologist, students | Neurological examination.  Review of medical records.  DSM-IV for dementia |

**Table 1** (continued)

| **Reference** | **Population**  **residence and habitat** | **No. individuals**  **(% response)*** | **Dementia,**  **no. cases** | **Age**  **(years)** | **Prevalence year** | **Screening phase**  **Instrument & Field workers** | **Diagnostic**  **ascertainment phase** |
| --- | --- | --- | --- | --- | --- | --- | --- |
| LEGANÉS (10) | Leganés (Madrid), suburban | 524  (Not specified) | 61 | > 70 | 1999 | Not formally screened. All individuals evaluated using MMSE, Short Portable Mental State Questionnaire (SPMSQ), 7-minute test, test of informant questionnaire, bell test, immediate and long-term recall, apprenticeship, trail-making test and WAIS subtest.  Field workers: 1 neurologist and 1 neuropsychologist. | All individuals underwent clinical evaluation  2 independent neurologists.  DSM-IV for dementia |
| PRATICON (17) | El Prat de Llobregat (Barcelona), suburban | 1,754  (85%) | 168 | ≥ 70 | 2002 | MMSE (Mini Mental State Examination). Cut off: < 24  Field workers: 21 trained psychologists | Neurologist and neuro-psychologist  DSM – IV for dementia |
| MUNGUIALDE (20) | Munguia (Vizcaya), urban-mixed | 1,931  (73.3%)***** | 175 | ≥ 65 | 2005 | MMSE (Mini Mental State Examination). Cut-off: 23/ 24  combined with Pfeffer SPMSQ. Cut-off: 4/5  Field workers: trained interviewers | Neurologists and geriatrician.  DSM-IV for dementia |

† Data refer to the total census sample obtained as registered screened survey population.
