# Supplementary material for: Prevalence of dementia and major dementia subtypes in Spanish populations: A reanalysis of dementia prevalence surveys, 1990-2008
Source: BMC Neurol. 2009 Oct 19;9:55. doi: 10.1186/1471-2377-9-55 (PMC2770986; doi:10.1186/1471-2377-9-55)
Supplement: Additional file 2 — Supplemental Table S2. Age and sex-specific counts for aged ≥ 70 years, crude, and age-adjusted or age- and sex-adjusted prevalence of dementia, Alzheimer's disease and vascular dementia in reanalyzed Spanish surveys. [file 1471-2377-9-55-S2.DOC]

**Table 2**: Age and sex-specific counts for aged ≥70 years, crude, and age-adjusted or age- and sex-adjusted prevalence of dementia, Alzheimer's disease and vascular dementia in reanalyzed Spanish surveys.

| **DEMENTIA by age- (years) and sex-groups** | | | | | | | | | | | | | | | | | | |
| --- | --- | --- | --- | --- | --- | --- | --- | --- | --- | --- | --- | --- | --- | --- | --- | --- | --- | --- |
| Survey | **70 – 74**  c^†^ / pop^‡^  P (%)^§^ | | | **75 – 79**  c / pop  P (%) | | | **80 – 84**  c / pop  P (%) | | | **85 – 89**  c / pop  P (%) | | | **> 89**  c / pop  P (%) | | | **70 and over**  c / pop  P (%) | | |
|  | **Both** | **Men** | **Women** | **Both** | **Men** | **Women** | **Both** | **Men** | **Women** | **Both** | **Men** | **Women** | **Both** | **Men** | **Women** | **Both^¶^** | **Men^††^** | **Women^††^** |
| Pamplona | 9 / 146  6.2 | 4 / 71  5.6 | 5 / 75  6.7 | 35 / 311  11.2 | 13 / 152  8.5 | 22 / 159  13.8 | 50 / 302  16.5 | 21 / 152  13.8 | 29 / 150  19.3 | 70 / 279  25.1 | 34 / 142  23.9 | 36 / 137  26.3 | 30 / 89  33.7 | 14 / 45  31.1 | 16 / 44  36.4 | 194 / 1,127  17.2 (12.0) | 86 / 562  15.3 (10.4) | 108 / 565  19.1 (13.6) |
| 1. **Munguialde** | 30 / 572  5.2 | 16 / 274  5.8 | 14 / 298  4.7 | 31 / 395  7.8 | 9 / 160  5.6 | 22 / 235  9.4 | 34 / 251  13.5 | 11 / 108  10.2 | 23 / 143  16.1 | 37 / 134  27.6 | 6 / 41  14.6 | 31 / 93  33.3 | 33 / 68  48.5 | 1 / 15  6.7 | 32 / 53  60.4 | 165 / 1,420  11.6 (10.2) | 43 / 598  7.2 (7.3) | 122 / 822  14.8 (13.0) |
| Gerona | 38 / 601  6.3 | 14 / 265  5.3 | 24 / 336  7.1 | 47 / 403  11.7 | 8 / 149  5.4 | 39 / 254  15.3 | 64 / 285  22.4 | 18 / 114  15.8 | 46 / 171  26.9 | 33 / 136  24.7 | 6 / 45  13.3 | 27 / 91  29.7 | 18 / 35  51.4 | 3 / 9  33.3 | 15 / 26  57.7 | 200 / 1460  13.7 (12.3) | 49 / 582  8.4 (8.4) | 151 / 878  17.2 (16.3) |
| 1. **Toledo** | 11 / 362  3.0 | 6 / 175  3.4 | 5 / 187  2.7 | 22 / 258  8.5 | 11 / 114  9.6 | 11 / 144  7.6 | 32 / 175  18.3 | 15 / 66  22.7 | 17 / 109  15.6 | 18 / 100  18.0 | 4 / 35  11.4 | 14 / 65  21.5 | 7 / 38  18.4 | 1 / 13  7.7 | 6 / 25  24.0 | 90 / 933  9.6 (8.8) | 37 / 403  9.2 (8.9) | 53 / 530  10.0 (8.7) |
| 1. **El Prat** | 21 / 749  2.8 | 11 / 344  3.2 | 10 / 405  2.5 | 29 / 492  5.9 | 11 / 212  5.2 | 18 / 280  6.4 | 40 / 280  14.3 | 15 / 109  13.8 | 25 / 171  14.6 | 40 / 151  26.5 | 9 / 46  19.6 | 31 / 105  29.5 | 38 / 82  46.3 | 10 / 24  41.7 | 28 / 58  48.3 | 168 / 1,754  9.6 (9.3) | 56 / 735  7.6 (8.6) | 112 / 1,019  11.0 (10.0) |
| 1. **Zarademp** | 18 / 837  2.1 | 4 / 345  1.1 | 14 / 492  2.8 | 14 / 617  2.3 | 5 / 245  2.0 | 9 / 372  2.4 | 34 / 480  7.1 | 8 / 188  4.2 | 26 / 292  8.9 | 79 / 635  12.4 | 25 / 269  9.3 | 54 / 366  14.7 | 69 / 281  24.5 | 12 / 78  15.4 | 57 / 203  28.1 | 214 / 2,850  7.5 (4.5) | 54 / 1,125  4.8 (3.1) | 160 / 1,725  9.3 (5.9) |
| 1. **Leganés** | 2 / 138  1.4 | 0 / 72  0.0 | 2 / 66  3.0 | 11 / 149  7.4 | 7 / 73  9.6 | 4 / 76  5.3 | 10 / 120  8.3 | 3 / 56  5.3 | 7 / 64  10.9 | 13 / 72  18.0 | 5 / 31  16.1 | 8 / 41  19.5 | 25 / 45  55.5 | 9 / 22  40.9 | 16 / 23  69.6 | 61 / 524  11.6 (8.4) | 24 / 254  9.4 (7.2) | 37 / 270  13.7 (9.6) |
| 1. **Zaragoza** | 9 / 301  3.0 | 7 / 135  5.2 | 2 / 166  1.2 | 11 / 255  4.3 | 7 / 99  7.1 | 4 / 156  2.6 | 17 / 157  10.8 | 9 / 58  15.5 | 8 / 99  8.1 | 11 / 82  13.4 | 4 / 26  15.4 | 7 / 56  12.5 | 6 / 20  30.0 | 1 / 6  16.7 | 5 / 14  35.7 | 54 / 815  6.6 (6.7) | 28 / 324  8.6 (8.7) | 26 / 491  5.3 (4.8) |
| 1. **Bidasoa** | 4 / 527  0.7 | 1 / 225  0.4 | 3 / 302  1.0 | 6 / 361  1.7 | 3 / 133  2.2 | 3 / 228  1.3 | 13 / 258  5.0 | 4 / 84  4.8 | 9 / 174  5.2 | 15 / 134  11.2 | 2 / 37  5.4 | 13 / 97  13.4 | 10 / 69  14.5 | 4 / 20  20.0 | 6 / 49  12.2 | 48 / 1,349  3.5 (3.2) | 14 / 499  2.8 (3.0) | 34 / 850  4.0 (3.4) |
| **ALZHEIMER’S DISEASE by age- (years) and sex-groups** | | | | | | | | | | | | | | | | | | |
| Survey | **70 – 74**  c / pop  P (%) | | | **75 – 79**  c / pop  P (%) | | | **80 – 84**  c / pop  P (%) | | | **85 – 89**  c / pop  P (%) | | | **> 89**  c / pop  P (%) | | | **70 and over**  c / pop  P (%) | | |
|  | **Both** | **Men** | **Women** | **Both** | **Men** | **Women** | **Both** | **Men** | **Women** | **Both** | **Men** | **Women** | **Both** | **Men** | **Women** | **Both** | **Men** | **Women** |
| Pamplona | 1 / 146  0.7 | 1 / 71  1.4 | 0 / 75  0.0 | 23 / 311  7.4 | 5 / 152  3.3 | 18 / 159  11.3 | 29 / 302  9.6 | 9 / 152  5.9 | 20 / 150  13.3 | 45 / 279  16.1 | 17 / 142  12.0 | 28 / 137  20.4 | 21 / 89  23.6 | 9 / 45  20.0 | 12 / 44  27.3 | 119 / 1,127  10.5 (6.3) | 41 / 562  7.3 (4.3) | 78 / 565  13.8 (8.2) |
| 1. **Munguialde** | 19 / 572  3.3 | 8 / 274  2.9 | 11 / 298  3.7 | 24 / 395  6.1 | 7 / 160  4.4 | 17 / 235  7.2 | 23 / 251  9.2 | 6 / 108  5.6 | 17 / 143  11.9 | 35 / 134  26.1 | 6 / 41  14.6 | 29 / 93  31.2 | 25 / 68  36.8 | 1 / 15  6.7 | 24 / 53  45.3 | 126 / 1,420  8.9 (7.7) | 28 / 598  4.7 (5.1) | 98 / 822  11.9 (10.4) |
| 1. **El Prat** | 16 / 601  2.7 | 5 / 265  1.9 | 11 / 336  3.3 | 17 / 403  4.2 | 1 / 149  0.7 | 16 / 254  6.3 | 28 / 285  9.8 | 9 / 114  7.9 | 19 / 171  11.1 | 10 / 136  7.3 | 0 / 45  0 | 10 / 91  11.0 | 10 / 35  28.6 | 2 / 9  22.2 | 8 / 26  30.8 | 81 / 1,460  5.5 (4.8) | 17 / 582  2.9 (2.6) | 64 / 878  7.3 (7.0) |
| 1. **Zarademp** | 4 / 362  1.1 | 1 / 175  0.6 | 3 / 187  1.6 | 14 / 258  5.4 | 5 / 114  4.4 | 9 / 144  6.2 | 17 / 175  9.7 | 8 / 66  12.1 | 9 / 109  8.2 | 13 / 100  13.0 | 3 / 35  8.6 | 10 / 65  15.4 | 4 / 38  10.5 | 0 / 13  0.0 | 4 / 25  16.0 | 52 / 933  5.6 (5.0) | 17 / 403  4.2 (4.1) | 35 / 530  6.6 (5.8) |
| 1. **Leganés** | 9 / 749  1.2 | 4 / 344  1.2 | 5 / 405  1.2 | 16 / 492  3.2 | 7 / 212  3.3 | 9 / 280  3.2 | 32 / 280  11.4 | 11 / 109  10.1 | 21 / 171  12.3 | 31 / 151  20.5 | 8 / 46  17.4 | 23 / 105  21.9 | 28 / 82  34.1 | 7 / 24  29.2 | 21 / 58  36.2 | 116 / 1,754  6.6 (6.4) | 37 / 735  5.0 (5.9) | 79 / 1,019  7.7 (6.9) |
| 1. **Zaragoza** | 7 / 837  0.8 | 0 / 345  0.0 | 7 / 492  1.4 | 7 / 617  1.1 | 1 / 245  0.4 | 6 / 372  1.6 | 27 / 480  5.6 | 4 / 188  2.1 | 23 / 292  7.9 | 49 / 635  7.7 | 16 / 269  5.9 | 33 / 366  9.0 | 41 / 281  14.6 | 10 / 78  12.8 | 31 / 203  15.3 | 131 / 2,850  4.6 (2.6) | 31 / 1,125  2.7 (1.5) | 100 / 1,725  5.8 (3.7) |
| 1. **Bidasoa** | 2 / 138  1.4 | 0 / 72  0.0 | 2 / 66  3.0 | 3 / 149  2.0 | 2 / 73  2.7 | 1 / 76  1.3 | 6 / 120  5.0 | 2 / 56  3.6 | 4 / 64  6.2 | 12 / 72  16.7 | 5 / 31  16.1 | 7 / 41  17.1 | 17 / 45  37.8 | 5 / 22  22.7 | 12 / 23  52.2 | 40 / 524  7.6 (5.3) | 14 / 254  5.5 (3.9) | 26 / 270  9.6 (6.7) |
| 1. **El Prat de Llobregat** | 7 / 301  2.3 | 5 / 135  3.7 | 2 / 166  1.2 | 6 / 255  2.3 | 2 / 99  2.0 | 4 / 156  2.6 | 17 / 157  10.8 | 9 / 58  15.5 | 8 / 99  8.1 | 7 / 82  8.5 | 3 / 26  11.5 | 4 / 56  7.1 | 5 / 20  25.0 | 1 / 6  16.7 | 4 / 14  28.6 | 42 / 815  5.1 (5.0) | 20 / 324  6.2 (6.1) | 22 / 491  4.5 (4.0) |

**Table 2** (continued)

| **VASCULAR DEMENTIA by age- (years) and sex-groups** | | | | | | | | | | | | | | | | | | |
| --- | --- | --- | --- | --- | --- | --- | --- | --- | --- | --- | --- | --- | --- | --- | --- | --- | --- | --- |
| Survey | **70 – 74**  c / pop  P (%) | | | **75 – 79**  c / pop  P (%) | | | **80 – 84**  c / pop  P (%) | | | **85 – 89**  c / pop  P (%) | | | **> 89**  c / pop  P (%) | | | **70 and over**  c / pop  P (%) | | |
|  | **Both** | **Men** | **Women** | **Both** | **Men** | **Women** | **Both** | **Men** | **Women** | **Both** | **Men** | **Women** | **Both** | **Men** | **Women** | **Both** | **Men** | **Women** |
| Pamplona | 4 / 146  2.7 | 1 / 71  1.4 | 3 / 75  4.0 | 6 / 311  1.9 | 3 / 152  2.0 | 3 / 159  1.9 | 7 / 302  2.3 | 4 / 152  2.6 | 3 / 150  2.0 | 4 / 279  1.4 | 4 / 142  2.8 | 0 / 137  0.0 | 5 / 89  5.6 | 2 / 45  4.4 | 3 / 44  6.8 | 26 / 1,127  2.3 (2.4) | 14 / 562  2.5 (2.0) | 12 / 565  2.1 (2.8) |
| 1. **Munguialde** | 8 / 572  1.4 | 6 / 274  2.2 | 2 / 298  2.0 | 3 / 395  0.8 | 0 / 160  0.0 | 3 / 235  1.3 | 8 / 251  3.2 | 3 / 108  2.8 | 5 / 143  3.5 | 2 / 134  1.5 | 0 / 41  0.0 | 2 / 93  2.2 | 3 / 68  4.4 | 0 / 15  0.0 | 3 / 53  5.7 | 24 / 1,420  1.7 (1.8) | 9 / 598  1.5 (1.3) | 15 / 822  1.8 (2.2) |
| 1. **El Prat** | 13 / 601  2.2 | 5 / 265  1.9 | 8 / 336  2.4 | 20 / 403  4.7 | 4 / 149  2.7 | 16 / 254  5.9 | 22 / 285  7.7 | 5 / 114  4.4 | 17 / 171  9.9 | 19 / 136  14.0 | 6 / 45  13.3 | 13 / 91  14.3 | 5 / 35  14.3 | 1 / 9  11.1 | 4 / 26  15.4 | 79 / 1,460  5.3 (5.1) | 21 / 582  3.6 (4.0) | 58 / 878  6.5 (6.2) |
| 1. **Zarademp** | 4 / 362  1.1 | 2 / 175  1.1 | 2 / 187  1.1 | 6 / 258  2.3 | 4 / 114  3.5 | 2 / 144  1.4 | 7 / 175  4.0 | 5 / 66  7.6 | 2 / 109  1.8 | 3 / 100  3.0 | 1 / 35  2.8 | 2 / 65  3.1 | 3 / 38  7.9 | 1 / 13  7.7 | 2 / 25  8.0 | 23 / 933  2.5 (2.4) | 13 / 403  3.2 (3.1) | 10 / 530  1.9 (1.8) |
| 1. **Leganés** | 8 / 749  1.1 | 6 / 344  1.7 | 2 / 405  0.5 | 4 / 492  0.8 | 1 / 212  0.5 | 3 / 280  1.1 | 3 / 280  1.1 | 1 / 109  0.9 | 2 / 171  1.2 | 3 / 151  2.0 | 1 / 46  2.2 | 2 / 105  1.9 | 3 / 82  3.6 | 1 / 24  4.2 | 2 / 58  3.4 | 21 / 1,754  1.2 (1.2) | 10 / 735  1.4 (1.4) | 11 / 1,019  1.1 (1.0) |
| 1. **Zaragoza** | 6 / 837  0.7 | 2 / 345  0.6 | 4 / 492  0.8 | 5 / 617  0.8 | 4 / 245  1.6 | 1 / 372  0.3 | 3 / 480  0.6 | 3 / 188  1.6 | 0 / 292  0.0 | 19 / 635  3.0 | 7 / 269  2.6 | 12 / 366  3.3 | 20 / 281  7.1 | 1 / 78  1.3 | 19 / 203  9.3 | 53 / 2,850  1.8 (1.2) | 17 / 1,125  1.5 (1.3) | 36 / 1,725  2.1 (1.2) |
| 1. **Bidasoa** | 0 / 138  0.0 | 0 / 72  0.0 | 0 / 66  0.0 | 4 / 149  2.7 | 2 / 73  2.7 | 2 / 76  2.6 | 4 / 120  3.3 | 1 / 56  1.8 | 3 / 64  4.7 | 1 / 72  1.4 | 0 / 31  0.0 | 1 / 41  2.4 | 3 / 45  6.7 | 3 / 22  13.6 | 0 / 23  0.0 | 12 / 524  2.3 (1.7) | 6 / 254  2.4 (1.8) | 6 / 270  2.2 (1.6) |
| Weights to adjust for European population | | | | | | | | | | | | | | | | | | |
|  | **70 – 74** | | | **75 – 79** | | | **80 – 84** | | | **>= 85** | | | | | | Total | | |
| Weights | 0.43 | | | 0.29 | | | 0.14 | | | 0.14 | | | | | | 1.00 | | |

Values for ‘85-89’ and ‘>89’ age levels were collapsed to ‘>84’ for European population adjustments.

† Cases; ‡ Pop: Population; §: P (%): Prevalence; ¶ Adjusted by age and sex (parenthetical data only); ††: Adjusted by age (parenthetical data only).
