# Supplementary material for: Prevalence of dementia and major dementia subtypes in Spanish populations: A reanalysis of dementia prevalence surveys, 1990-2008
Source: BMC Neurol. 2009 Oct 19;9:55. doi: 10.1186/1471-2377-9-55 (PMC2770986; doi:10.1186/1471-2377-9-55)
Supplement: Additional file 3 — Supplemental Table S3. Prevalence OR of dementia, Alzheimer's disease and vascular dementia with 95% CI, adjusted for age and age- and sex (reference: El Prat survey). [file 1471-2377-9-55-S3.DOC]

**Table 3:** Prevalence OR of dementia, Alzheimer's disease and vascular dementia with 95% CI, adjusted for age and age- and sex (reference: El Prat survey).

|  | **MEN** | | | **WOMEN** | | | **BOTH** | | |
| --- | --- | --- | --- | --- | --- | --- | --- | --- | --- |
|  | **Dementia** | **Alzheimer's disease** | **Vascular dementia** | **Dementia** | **Alzheimer’s disease** | **Vascular dementia** | **Dementia** | **Alzheimer’s disease** | **Vascular dementia** |
| 1. **Pamplona** | 1.28 (0.88 – 1.86) | 0.74 (0.46 – 1.20) | 1.32 (0.58 – 3.03) | 1.28 (0.94 – 1.75) | 1.26 (0.89 – 1.79) | 1.49 (0.64 – 3.49) | 1.28 (1.01 – 1.62) | 1.06 (0.79 – 1.41) | 1.42 (0.79 – 2.58) |
| 1. **Munguialde** | 0.92 (0.60 – 1.41) | 0.90 (0.53 – 1.52) | 1.09 (0.44 – 2.71) | 1.38 (1.04 – 1.84) | 1.58 (0.14 – 2.18) | 1.63 (0.74 – 3.59) | 1.21 (0.96 – 1.53) | 1.35 (1.03 – 1.77) | 1.39 (0.77 – 2.51) |
| 1. **Gerona** | 1.10 (0.73 – 1.66) | 0.54 (0.30 – 0.99) | 2.69 (1.25 – 5.75) | 1.94 (1.47 – 2.56) | 1.01 (0.70 – 1.43) | 7.26 (3.75 – 14.05) | 1.61 (1.28 – 2.02) | 0.85 (0.63 – 1.15) | 5.00 (3.06 – 8.16) |
| 1. **Toledo** | 1.16 (0.74 – 1.82) | 0.75 (0.41 – 1.39) | 2.34 (1.02 – 5.38) | 0.85 (0.59 – 1.22) | 0.79 (0.51 – 1.21) | 1.74 (0.73 – 4.16) | 0.96 (0.72 – 1.28) | 0.78 (0.55 – 1.11) | 2.05 (1.12 – 3.73) |
| 1. **El Prat** | 1 | 1 | 1 | 1 | 1 | 1 | 1 | 1 | 1 |
| 1. **ZARADEMP** | 0.39 (0.26 – 0.58) | 0.30 (0.18 – 0.50) | 0.87 (0.39 – 1.92) | 0.52 (0.40 – 0.68) | 0.47 (0.34 – 0.65) | 1.45 (0.74 – 2.85) | 0.48 (0.38 – 0.60) | 0.42 (0.32 – 0.55) | 1.18 (0.71 – 1.95) |
| 1. **Leganés** | 0.89 (0.54 – 1.47) | 0.70 (0.37 – 1.32) | 1.43 (0.52 – 3.91) | 0.98 (0.65 – 1.46) | 0.96 (0.60 – 1.53) | 1.71 (0.61 – 4.75) | 0.93 (0.68 – 1.28) | 0.87 (0.60 – 1.27) | 1.57 (0.76 – 3.23) |
| 1. **Zaragoza** | 1.12 (0.69 – 1.82) | 1.21 (0.68 – 2.17) | – | 0.45 (0.29 – 0.70) | 0.56 (0.34 – 0.92) | – | 0.66 (0.47 – 0.91) | 0.76 (0.52 – 1.10) | – |
| 1. **Bidasoa** | 0.32 (0.17 – 0.57) | – | – | 0.29 (0.19 – 0.43) | – | – | 0.29 (0.21 – 0.41) | – | – |
|  |  |  |  |  |  |  |  |  |  |
| **Men** | – | – | – | – | – | – | 1 | 1 | 1 |
| **Women** | – | – | – | – | – | – | 1.45 (1.27 – 1.66) | 1.73 (1.45 – 2.06) | 1.09 (0.84 – 1.43) |
|  |  |  |  |  |  |  |  |  |  |
| **70 – 74** | 1 | 1 | 1 | 1 | 1 | 1 | 1 | 1 | 1 |
| **75 – 79** | 1.66 (1.18 – 2.34) | 1.78 (1.03 – 3.06) | 1.18 (0.63 – 2.20) | 2.11 (1.59 – 2.82) | 2.39 (1.63 – 3.50) | 1.82 (1.03 – 3.21) | 1.91 (1.53 – 2.38) | 2.17 (1.59 – 2.96) | 1.51 (0.99 – 2.29) |
| **80 – 84** | 3.56 (2.56 – 4.95) | 5.34 (3.27 – 8.72) | 2.03 (1.11 – 3.71) | 4.74 (3.60 – 6.26) | 5.46 (3.79 – 7.88) | 2.79 (1.58 – 4.92) | 4.18 (3.39 – 5.16) | 5.35 (3.99 – 7.17) | 2.39 (1.58 – 3.60) |
| **85 – 89** | 5.30 (3.76 – 7.46) | 8.84 (5.41 – 14.43) | 2.62 (1.38 – 4.99) | 8.60 (6.49 – 11.38) | 9.46 (6.58 – 13.60) | 3.97 (2.21 – 7.13) | 7.09 (5.72 – 8.78) | 9.09 (6.79 – 12.17) | 3.28 (2.14 – 5.04) |
| **> 89** | 10.18 (6.77 – 15.3) | 16.67 (9.65–28.78) | 3.77 (1.70 – 8.39) | 20.66 (15.25-27.99) | 19.79 (13.48 – 29.05) | 10.27 (5.70 – 18.49) | 16.19 (12.78 – 20.53) | 18.58 (13.61 – 25.37) | 7.24 (4.62 – 11.35) |
