# Supplementary material for: Prevalence of dementia and major dementia subtypes in Spanish populations: A reanalysis of dementia prevalence surveys, 1990-2008
Source: BMC Neurol. 2009 Oct 19;9:55. doi: 10.1186/1471-2377-9-55 (PMC2770986; doi:10.1186/1471-2377-9-55)
Supplement: Additional file 4 — Supplemental Table S4. Age- and sex-adjusted prevalence odd ratios plus 95% confidence interval of dementia, Alzheimer's disease and vascular dementia for selected factors among ≥ 70 participants. [file 1471-2377-9-55-S4.DOC]

**Table 4:** Age- and sex-adjusted prevalence odd ratios plus 95% confidence interval of dementia, Alzheimer’s disease and vascular dementia for selected factors among ≥70 participants.

| **Selected comparisons^†^** | **MEN** | | | **WOMEN** | | | **BOTH** | | |
| --- | --- | --- | --- | --- | --- | --- | --- | --- | --- |
|  | **Dementia** | **Alzheimer’s disease** | **Vascular dementia** | **Dementia** | **Alzheimer’s disease** | **Vascular dementia** | **Dementia** | **Alzheimer’s disease** | **Vascular dementia** |
| **Model I** |  |  |  |  |  |  |  |  |  |
| Neurologists | 1 | 1 | 1 | 1 | 1 | 1 | 1 | 1 | 1 |
| Psychiatrists | 0.54 (0.41 – 0.70) | 0.56 (0.40 – 0.79) | 0.59 (0.34 – 1.03) | 0.47 (0.39 – 0.56) | 0.41 (0.33 – 0.52) | 0.58 (0.40 – 0.84) | 0.49 (0.42 – 0.57) | 0.45 (0.38 – 0.55) | 0.59 (0.44 – 0.81) |
| Geriatricians | 1.22 (0.84 – 1.78) | 0.95 (0.56 – 1.63) | 1.59 (0.86 – 2.93) | 0.77 (0.56 – 1.06) | 0.67 (0.46 – 0.98) | 0.67 (0.35 – 1.30) | 0.92 (0.72 – 1.18) | 0.75 (0.55 – 1.02) | 1.00 (0.64 – 1.57) |
| **Model II** |  |  |  |  |  |  |  |  |  |
| Urban | 1 | 1 | 1 | 1 | 1 | 1 | 1 | 1 | 1 |
| Suburban | 1.19 (0.91 – 1.57) | 1.65 (1.16 – 2.36) | 0.80 (0.45 – 1.41) | 1.13 (0.92 – 1.39) | 1.40 (1.09 – 1.78) | 0.43 (0.26 – 0.73) | 1.16 (0.98 – 1.36) | 1.47 (1.20 – 1.80) | 0.56 (0.38 – 0.81) |
| Urban-mixed | 0.94 (0.73 – 1.22) | 1.54 (1.06 – 2.22) | 1.13 (0.68 – 1.87) | 0.89 (0.74 – 1.07) | 1.75 (1.39 – 2.21) | 0.62 (0.39 – 0.96) | 0.91 (0.79 – 1.06) | 1.69 (1.39 – 2.06) | 0.79 (0.56 – 1.10) |
| **Model III** |  |  |  |  |  |  |  |  |  |
| Collaboration ≥ 85% | 1 | 1 | 1 | 1 | 1 | 1 | 1 | 1 | 1 |
| Collaboration < 85% | 0.62 (0.48 – 0.81) | 0.76 (0.53 – 1.08) | 0.68 (0.41 – 1.14) | 0.45 (0.38 – 0.54) | 0.73 (0.58 – 0.91) | 0.42 (0.29 – 0.59) | 0.50 (0.43 – 0.58) | 0.74 (0.61 – 0.89) | 0.50 (0.37 – 0.66) |

†: Reference category listed first in all models.
